# Uterine dehiscence in pregnant with previous caesarean delivery

**DOI:** 10.1080/07853890.2021.1959049

**Published:** 2021-07-26

**Authors:** Zhengfeng Zhu, HeZhou Li, JunQing Zhang

**Affiliations:** a Ultrasonic Department, Zhengzhou University Third Hospital and Henan Province Women and Children's Hospital, Zhengzhou, China; b Medical Administrative Department, Zhengzhou University Third Hospital and Henan Province Women and Children's Hospital, Zhengzhou, China

**Keywords:** Caesarean scar, pregnancy, ultrasound, uterine scar dehiscence

## Abstract

**Background:**

The main risk factor for uterine scar dehiscence is a previous caesarean section. Better characterisation of the ultrasonographic features of uterine scar dehiscence may improve preoperative diagnostic accuracy in pregnant women with a caesarean scar. This study aimed to evaluate the ultrasonographic features of uterine scar dehiscence in pregnant women and maternal and neonatal outcomes.

**Materials and methods:**

This was a retrospective review of the records of 23 women with a previous caesarean section found to have uterine scar dehiscence during surgery. The integrity and thickness of the lower uterine segment were recorded, ultrasonographic features were evaluated, and maternal and infant outcomes were analysed.

**Results:**

Of the 23 cases of uterine scar dehiscence, six were detected by preoperative ultrasonography, while 17 were missed. The ultrasonographic features of the 23 cases of uterine dehiscence included anechoic areas protruding through the caesarean section scar with an intact serosal layer (4/23), disappearance of the muscular layer (2/23), and a thinner lower uterine segment (17/23). There were no cases of maternal or neonatal mortality. One woman chose to undergo pregnancy termination.

**Conclusion:**

Preoperative detection of uterine scar dehiscence in women with previous caesarean delivery helps prevent maternal and neonatal morbidity and mortality. However, the maximum benefit can only be obtained by scanning at appropriate intervals during pregnancy and accurate recognition of the ultrasonographic features of uterine scar dehiscence.

**KEY MESSAGES**
1. Preoperative detection of uterine scar dehiscence in women with previous caesarean delivery helps prevent maternal and neonatal morbidity and mortality.
2. Scanning at appropriate intervals during pregnancy and accurate recognition of the ultrasonographic features of uterine scar dehiscence could be beneficial.
3. Even when uterine dehiscence is detected by ultrasound during the second trimester, conservative management via strict observation alone is also feasible.

## Introduction

Uterine scar dehiscence is a common complication of caesarean delivery, which increases the risk of uterine rupture [1]. Uterine rupture is a complete division of all three layers of the uterus: the perimetrium, myometrium, and endometrium; while uterine dehiscence is considered an incomplete division of the three layers, allowing visibility of the foetus through the perimetrium. Uterine dehiscence is often asymptomatic [2]. The caesarean section rate worldwide is high; in Shanghai ofChina, the overall caesarean section rate was reported to be 41.5% [3]. Over the last five decades in the United States, the caesarean section rate has increased mainly between 1970 and 2016 [4]. Cases of underreporting do occur; in one study, the diagnosis of complete uterine rupture was underreported by 35% in their electronic patient record system (EPRS), and diagnosis of uterine dehiscence was missing in 100% of cases [5].

Therefore, this study aimed to evaluate the ultrasonographic features of uterine scar dehiscence in pregnant women and the maternal and neonatal outcomes to enable early diagnosis and appropriate clinical management.

## Material and methods

This was a retrospective study of uterine scar dehiscence in pregnant women with previous caesarean delivery at the Third Affiliated Hospital of Zhengzhou University, a tertiary centre of obstetrics and gynaecology. Data on deliveries that occurred between January 2016 and December 2020 were retrieved from the hospital’s EPRS. The study was approved by the Institutional Review Board of the Zhengzhou University Third Hospital. Written consent was not obtained owing to the retrospective nature of the study. Transabdominal ultrasound scanning was performed using a GE Voluson E8 with an abdominal 1–5 MHz probe (GE Healthcare, Kretztechnik, Zipf, Austria).

The inclusion criteria were as follows: all pregnant women with a history of caesarean delivery, who underwent ultrasound assessment at least twice (once in the second trimester and once in the third trimester) at the center before surgery.

Exclusion criteria were as follows: Presence of foetal anomalies, malpresentation, abnormal amniotic fluid volume, suspected placental abruption, previous uterine surgery other than caesarean section, and women with confirmed or suspected placenta accreta or previa.

Experienced ultrasonographers checked the integrity of the uterine scar during the second trimester and measured the thickness of the lower uterine segment scar during the third trimester. Ultrasonography was performed with a partially full bladder, and the lower uterine segment scar thickness was visualised in the sagittal plane under magnification to measure the thinnest muscularis area [6]. The diagnosis of uterine scar dehiscence was confirmed during surgery when separation of the lower uterine segment scar and the foetus was visible through the peritoneum.

## Results

Twenty-three women were diagnosed with uterine scar dehiscence. Twenty-two cases were singletons and one was a twin. The median age was 34 years (range, 23–40 years). Fourteen women had undergone previous caesarean section, while nine women had undergone two previous caesarean sections. Twenty women underwent elective caesarean sections; one underwent an emergency caesarean section because of worsening abdominal pain; one underwent an emergency caesarean section following a failed vaginal delivery trial, foetal bradycardia, and gross haematuria per indwelling catheter; and one underwent vaginal delivery.

The preoperative ultrasonographic detection rate of uterine scar dehiscence was 26.1% (6/23), as confirmed by caesarean section. The intraoperative detection rate was 69.6% (16/23), and the postpartum detection rate was 4.3% (1/23)based on clinical symptoms and physical examination.

**Figure 1.**
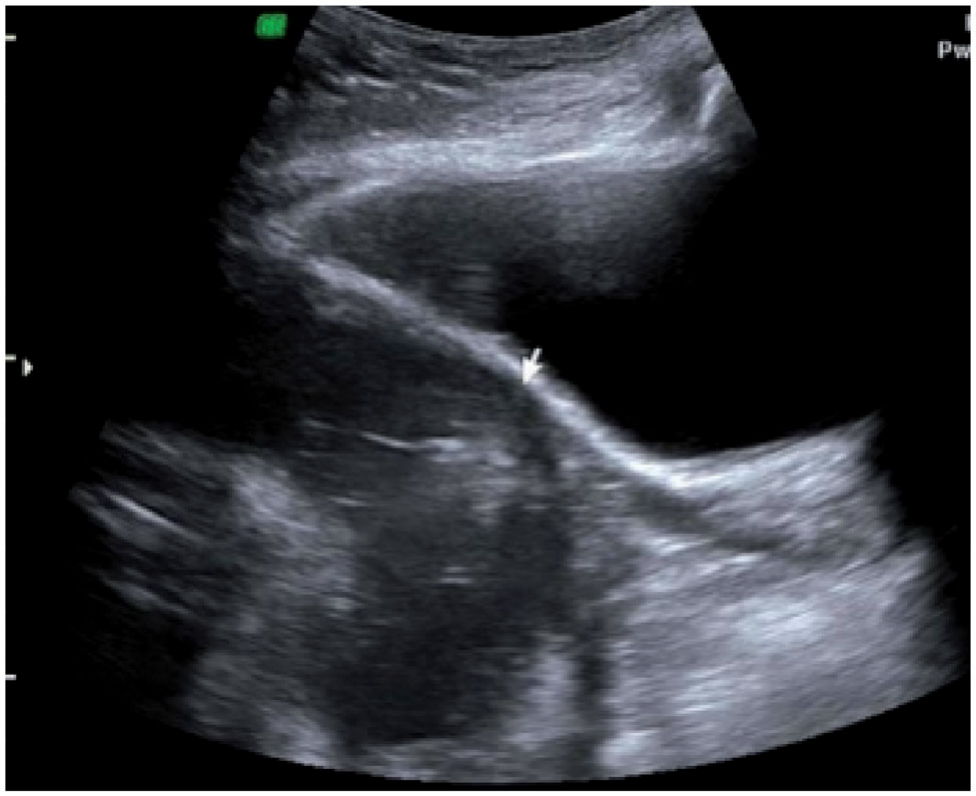
Absence of the muscle layer in the lower uterine segmentat the area of the caesarean section scar.

**Figure 2.**
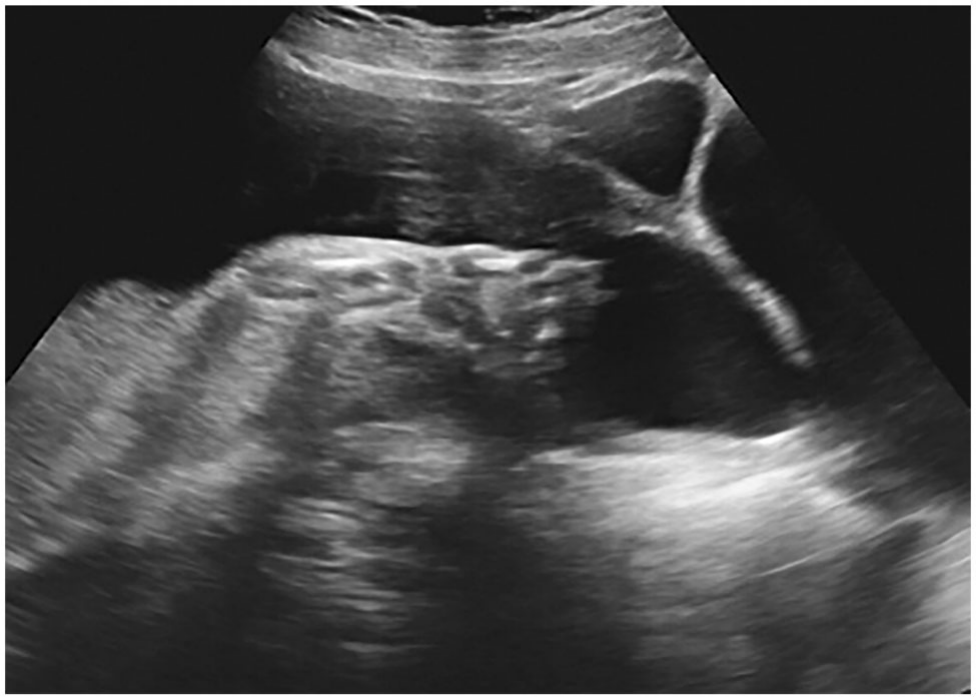
An anechoic area in the lower uterine segment at the area of the caesarean section scar.

Regarding the integrity and thickness of the lower uterine segment scar from the previous caesarean section, the absence of the muscular layer was detected *via* ultrasound in two patients (Figure 1). For these two cases, one was diagnosed at 29 weeks and opted for observation until 33 weeks, while the other patient was diagnosed at 33 weeks and chose to undergo immediate caesarean delivery. An anechoic area bulging in the area of the lower segment scar was detected *via* ultrasound in four patients (Figure 2). Among these four cases, one was diagnosed at 27 weeks and chose to terminate the pregnancy, one was diagnosed at 24 weeks and opted for observation until 37 weeks, one was diagnosed at 26 weeks and opted for observation until 34 weeks, and one was diagnosed at 37 weeks and chose to undergo immediate caesarean delivery. A scar thickness between 1.0 and 1.8 mm was detected intraoperatively in 16 women with gestational ages between 37 and 40 weeks. In the case of the woman who was found to have uterine scar dehiscence after vaginal delivery, the thickness of the lower uterine segment caesarean section scar was 1 mm at 38 weeks of gestation.

Twenty-two of our cases of uterine scar dehiscence underwent repeat elective caesarean section. In some cases with local weakness or missing lower uterine segment, trimming and reinforcement of the scar tissue incision was made with two layers of continuous, absorbable sutures. In one case of uterine scar dehiscence after vaginal delivery, the patient was managed conservatively, including anaemia correction, bladder irrigation, other symptomatic treatment, and hospitalisation with close monitoring.

## Discussion

This study showed that most cases of uterine scar dehiscence were detected intraoperatively, in agreement with previous studies [7,8]. Diagnosing uterine scar dehiscence after vaginal delivery is challenging because of its vague presentation. The main ultrasonographic diagnostic features of uterine scar dehiscence are the absence of the uterine muscle layer and an anechoic area protruding through the lower segment caesarean section scar with an intact serosal layer. Uterine scar dehiscence was expected to be more common in women who have a history of >1 caesarean deliveries, but this complication was found in 14 women with only one caesarean section as opposed to nine who had two. However, the previous history of caesarean section, the characteristics (i.e. number of layers and the kind of thread, etc.) of the previous uterine breach sutures, and trimming and reinforcement of scar tissue during the second caesarean section, might be factors that were lacking in these records.

In this study, the lower uterine segment thickness, measured by transabdominal ultrasound, was between 1.0and 1.8 mm at term. It is known that the degree of thinning of the lower uterine segment is related to the risk of a defective scar and the risk of uterine rupture. Rozenberg et al. evaluated the usefulness of ultrasonographic measurement of the lower uterine segment before labour in predicting the risk of intrapartum uterine rupture. They found that with a cut-off value of 3.5 mm, the sensitivity of ultrasonographic measurement in predicting the risk of intrapartum uterine rupture was 88%, the specificity was 73.2%, the positive predictive value (PPV) was 11.8%, and the negative predictive value (NPV) was 9.3%. They also concluded that the risk of a defective scar is directly related to the degree of thinning of the lower uterine segment at approximately 37 weeks of pregnancy [9]. Qureshi et al. evaluated the lower uterine segment thickness using transvaginal ultrasonography to predict the integrity and quality of caesarean scars during pregnancy. They found that ultrasonographic evaluation of the lower uterine segment correlated well with operative findings and effectively predicted the quality of the uterine scar. A lower uterine segment thickness of > 2 mm effectively differentiated the risk group of potential uterine rupture from the non-risk group, as a lower uterine segment thickness of less than 2 mm was considered a criterion for poor healing, with a sensitivity and specificity of 86.7% and 100%, respectively, a PPV of 100%, and an NPV of 86.7%[10].

In our study, uterine scar dehiscence was primarily diagnosed during cesarean delivery, except in three cases diagnosed during the second trimester. One case was diagnosed during a laparotomy following a vaginal delivery trial, and one case was diagnosed following a successful vaginal delivery, similar to the case in the literature [11]. All outcomes were favourable.

It showed that even when uterine dehiscence was detected by ultrasound in the second trimester, conservative management *via* strict observation was also feasible. There was no maternal mortality in our study, which might be due to appropriate management, and was similar to the outcomes obtained by Fox et al. and Hamar et al. [12,13].

Ultrasonographic evaluation of the lower uterine segment is a non-invasive, reproducible, and safe technique for estimating the risk of uterine scar dehiscence and rupture. It is a strong predictor of uterine scar defects in women with a previous caesarean section [14,15]. It has been suggested that the risk of recurrent uterine ruptures can be reduced by elective caesarean section at 36–37 weeks gestational age, in women with a history of isthmic rupture, and at 32–33 weeks gestational age after foetal lung maturation in women with a history of fundal uterine rupture and those with short inter-pregnancy intervals [16]. However, there is currently no gold standard for diagnosing caesarean scar dehiscence [11].

Indeed, it is increasingly common to find pregnant women who have previously undergone myomectomy, who also have a considerable risk of uterine rupture. Recently, an interesting paper was published regarding the possible role of barbed sutures during laparoscopic myomectomy on pregnancy outcomes [17]. Whether the role of different types of suturing techniques play a role in uterine scar dehiscence, in both cesarean section and myomectomy, requires further study. In this study, conservative management *via* observation was feasible in the early third trimester, and repeated caesarean section was safer and more effective when ultrasonographic diagnosis of uterine dehiscence was evaluated. Similar cut-offs were also reported in a very large meta-analysis addressing the risk of uterine rupture or dehiscence during a trial of labour after caesarean section [18].

There were some limitations to this study. The data obtained for patients with uterine dehiscence were retrieved from the electronic patient record system between January 2016 and December 2020. This is because during this period, our hospital’s ultrasound department acquired the technology and clinical capability to diagnose uterine scar dehiscence and had great progress compared to previous years.No major adverse outcomes occurred during the study period, which may reflect good management of obstetric complications and may also be attributed to the small number of cases included in the study. Furthermore, there are other factors related to uterine dehiscence, which should be further explored in subsequent studies. Whether measurement in the second and third trimesters is sufficient would also require further studies. Lastly, uterine rupture outside of labour is a very rare complication following caesarean section and is not part of the scope of our study. Further studies with a large number of patients are required in order to explore these cases.

## Conclusions

Most cases of uterine scar dehiscence are diagnosed at the time of repeat caesarean section for maternal or foetal indications with occasional diagnoses following vaginal delivery in women with previous caesarean delivery. Diagnosis by ultrasonographic assessment of the lower uterine segment, especially in the second trimester, is helpful, but there is currently no gold standard for the diagnosis. A high index of suspicion, appropriate diagnostic modalities, and case management are essential for good maternal and neonatal outcomes.

## Authors’ contributions

ZhengFeng Zhu was involved in the conception and design, and the drafting of the paper. JunQing Zhang was involved in the analysis and interpretation of the data. HeZhou Li was involved in revising it critically for intellectual content; ZhengFeng Zhu and HeZhou Li were involved in the final approval of the version to be published; and that all authors agree to be accountable for all aspects of the work.

## Data Availability

The authors confirm that the data supporting the findings of this study are available within the article.
